# Cutaneous Leishmaniasis in Algeria; Highlight on the Focus of M’Sila

**DOI:** 10.3390/microorganisms9050962

**Published:** 2021-04-29

**Authors:** Razika Benikhlef, Karim Aoun, Abdelkarim Boudrissa, Meriem Ben Abid, Kamel Cherif, Wafa Aissi, Souad Benrekta, Said C. Boubidi, Gerald F. Späth, Aïda Bouratbine, Denis Sereno, Zoubir Harrat

**Affiliations:** 1 Laboratoire d’Eco-épidémiologie Parasitaire et Génétique des Populations, Route du Petit Staoueli, Institut Pasteur d’Algérie, Dely-Brahim 16047, Algeria; saidboubidi@yahoo.fr (S.C.B.); harrat_zoubir@yahoo.fr (Z.H.); 2 Laboratoire de Recherche “Parasitologie Médicale, Biotechnologies et Biomolécules”, LR 20-IPT-06, Institut Pasteur de Tunis, Université Tunis El Manar, 13, Pl Pasteur BP 74, Tunis 1002, Tunisia; karim.aoun@pasteur.tn (K.A.); meriembenabid@gmail.com (M.B.A.); aida.bouratbine@pasteur.tn (A.B.); 3 Laboratoire d’Epidémiologie et d’Ecologie Parasitaires, Institut Pasteur de Tunis, 13, Pl Pasteur BP 74, Tunis 1002, Tunisia; 4 Antenne de M’Sila, Institut Pasteur d’Algérie, M’sila 28000, Algeria; boudrissakarim@yahoo.fr; 5 Département de Biologie, Université de M’Sila, BP 166, M’Sila 28000, Algeria; kamel.cherif@univ-msila.dz; 6 Centre National pour la Promotion de la Transplantation d’Organes, Faculté de Médecine de Tunis, Université Tunis El Manar, Rue Jebel Lakdhar, Tunis 1006, Tunisia; wafa.aissi@fmt.utm.tn; 7 Service d’Epidémiologie et de Médecine Préventive, Commune de Boussaâda, M’sila 280001, Algeria; souadbenrekta@gmail.com; 8 Unité de Parasitologie Moléculaire et Signalisation (ParSig), Department of Parasites and Insect Vectors (PIV), Institut Pasteur/INSERM U1201, 25 Rue du Dr. Roux, 75015 Paris, France; gerald.spaeth@pasteur.fr; 9 IRD, University of Montpellier, InterTryp, 34000 Montpellier, France; 10 IRD, University of Montpellier, MiVeGec, 34000 Montpellier, France

**Keywords:** cutaneous leishmaniasis, epidemiology, *Leishmania major*, sandflies, rodents, M’Sila, Algeria

## Abstract

Algeria ranks second after Afghanistan for the incidence of cutaneous leishmaniasis (CL) worldwide. Here, we report a 34-years retrospective analysis of CL in Algeria and focused on the most affected region, the M’Sila province. All 66 cutaneous isolates corresponded to *Leishmania* (*L*.) *major*. Our study of the sandfly and rodent fauna further highlighted the high density of *Phlebotomus papatasi* and additional phlebotomine species of medical importance, not previously identified in M’Sila. Wild rodents belonging to nine species were trapped in M’Sila, and *Psammomys obesus* and *Meriones shawi* were found infected by *L. major*. In addition, *Leishmania infantum* was isolated from two visceral leishmaniasis cases, one dog and its proven vectors (*P. perniciosus*, *P. longicuspis*, and *P. perfiliewi*) inventoried during the survey. The high incidence of CL in the M’Sila province is likely a consequence of the increase in minimum temperatures recorded that constitutes suitable conditions for establishing a high endemicity and leads to an explosive rise in leishmaniases cases in this region. A thorough investigation of the underlying risk factors is urgently needed to detect new cases earlier. All these would improve the preparedness to fight the disease.

## 1. Introduction

Algeria ranks second after Afghanistan in cutaneous leishmaniasis (CL) incidence [1]. Initially confined in the high plateaus’ steppe regions in the semi-arid and arid bioclimatic zones [2,3], CL has undergone a worrying geographic extension toward the north of Algeria [4]. Such an expansion also occurs in the Maghreb and the Middle East [1,5,6,7].

**Figure 1:**
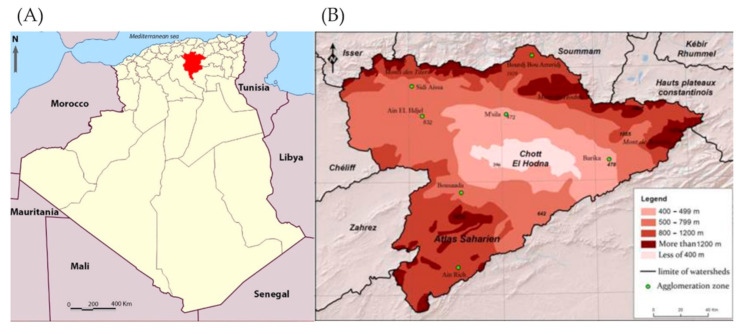
Geographic location of (**A**) the M’Sila province and (**B**) the salt-lake of Chott el Hodna [19].

The province of M’Sila is located in Central-Eastern Algeria and is most affected by the disease, with nearly 90,000 cumulative cases recorded since 1982 [8]. Cutaneous leishmaniasis is first described in 1860 in Algeria [9] and 1924 in the Hodna basin [10]. Since the 1980s, the situation has become alarming, with about 3000 annual cases [11]. Zoonotic CL (ZCL), caused by *L. major*, is currently the only clinical form endemic in the region [12]. The wild rodents *Psammomys* (*P*). *obesus* and *Meriones* (*M*). *shawi* are the primary reservoirs, and *Phlebotomus* (*P*). *papatasi* the proven vector [13,14,15]. The province of M’Sila is an agro-pastoral area with 1.3 million inhabitants over 18,175 km^2^ [16]. It is located 240 km south-east of Algiers, in the country’s highlands within the broad semi-arid and arid region of Hodna (Figure 1A,B). The Hodna region is a vast, steppe-like plain, silty in the North and sandy in the South, with four distinct areas, i.e., mountains, piedmont, lowland, and the Sebkha, a flooded grassland (76,000 hectares of bare clay) [17]. The hydro-geographic basin of Hodna is the most active focus of CL, with more than 75% of cases identified in this basin [18]. Seven provinces (M’Sila, Batna, Bordj-Bou Arreridj, Djelfa, Médéa, Bouira, and Sétif), belonging to the same noso-geographical region, are linked together by a tangled hydrographic network and a dense road network (Figure 1A,B). Biskra, the former and still active focus of ZCL, is only 190 km from M’Sila city (Figure 1A).

Starting from a longitudinal survey on the incidence of CL cases from 1982 to 2020 in the M’Sila focus, we gathered data on the diversity of the phlebotomine sandfly and rodent fauna and ecological factors in this region. We put them in perspective with the overall CL epidemiological situation in Algeria. These analyses are helpful to improve future public health strategies aimed at controlling ZCL.

## 2. Materials and Methods

### 2.1. Study Area and Study Design

**Figure 2:**
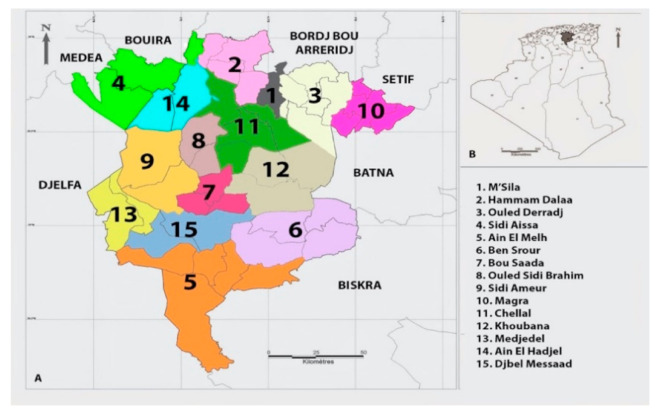
Administrative districts (Daïras) of the M’Sila province.

The province of M’Sila locates between 35°18′and 35°32′ North and 4°15′and 5°06′ East, 240 km south-east of Algiers, in the country’s highlands. Rainfall varies from 69 mm to 200 mm/year, and the average annual temperatures between 13 °C and 23 °C, with extremes of −5 °C in winter to 46 °C in summer [20]. The province encompasses 15 Daïras (districts), and the most populated one is M’Sila, a city followed by Boussaâda, called “the gateway to the Sahara” (Figure 2).

In the endemic foci of M’Sila, we collected data on ZCL incidence from 1982 to 2020 and other associated information, including clinical aspects of lesions, the dynamic and diversity of the phlebotomine, and the diversity of the wild rodent’s population. In parallel, data on CL incidence in Algeria were computed for the 48 provinces of the country from 1982 to 2017 [8]. A phlebotomine fauna survey was carried out from 2003 to 2004 and in 2018 in various sites: stables, sheepfolds, gardens, rodent burrows, palm groves, crevices, and dwellings (Table 1).

Rodents were trapped from 1996 to 2018 to determine the wild rodent population diversity. A prospective study on suspected CL and other suspected human and canine visceral leishmaniasis was carried out from October 2018 to February 2019.

### 2.2. Entomological Sampling

**Figure 3:**
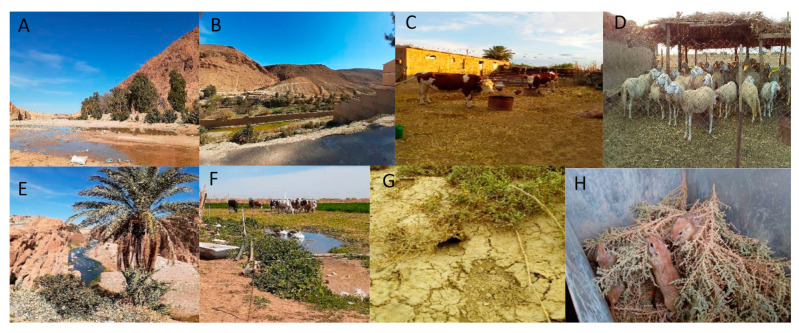
Pictures depicting the diversity of the ecological environment of the trapping station. ((**A**,**B**,**E**) (Bou Saâda), (**C**,**D**), (Ait Ikhlef, Chellal), (**F**) (Ain El Hadjel), (**G**,**H**) (Ouled Madhi, Chellal)).

Sandflies were collected from March to October 2003/2004 during the active season of transmission in 13 stations from 9 districts (Figure 3).

In five stations (Nouara, Ouled Madhi, Maadid, Ain El Hadjel, Boussaâda), the trapping effort was carried out twice a month over the transmission season (March to October). The other trapping sites were sampled one time along east-west and north-south transects. Sandflies were collected using adhesive paper (20 cm × 20 cm papers soaked in castor oil) and CDC light traps (John W., Hock Co., Gainesville, FL, USA) as described by Rioux et al. [21]. Specimens were collected and stored in glass vials containing 70% ethanol for morphological identification, using dichotomous keys of Abonnenc [22], Croset et al. [23], and Dedet et al. [24]. Seasonal fluctuation of the sandfly population was ascertained in the station of Ain El Hadjel (Table 1).

### 2.3. Rodent Capture

Trapping of rodents was performed during 1996, 2003, 2004, 2007, 2010, 2012, and 2018, based on indications of their presence (burrows, fragments of Chenopodiaceae plants, droppings, and traces of urine). Sherman traps were placed along a transect of the salt-lake (sebkha) and down the hills’ piedmont (Table 2).

Rodents were anesthetized with carbon monoxide (CO); morphological identification was performed according to identification keys described by Bernard [25]. Auricle, muzzle, legs, and tail exposed to insect bites were carefully examined. A biopsy of lesions was taken and smeared on a glass slide and stained with Giemsa. A portion of the sample was inoculated into an NNN medium supplemented with few Penicillin antibiotic drops (1U/mL) solution under sterile conditions [26,27].

The spleen was collected and homogenized in microtubes containing PBS with single-use pellet pestles (Polylabo, France). Aliquots of the suspension were stored at −20 °C until DNA extraction. According to the manufacturer protocol, the total DNA was purified using the QIAamp DNA mini kit (QIAGEN). Parasite DNA was detected by PCR amplification of the ribosomal internal transcribed spacer 1 (ITS1), using LITSR and L5.8S primers [28].

### 2.4. Patient: Skin Wound Sampling Collection

Ninety-six patients with evocative CL lesions were recruited during a routine diagnosis of the disease. Sixty patients were from Boussaâda and 36 from M’Sila city. Skin lesions were scarified using a curved sterile scalpel blade (Swann-Morton-Sinner, France), as described in the WHO guidelines for diagnosing human CL [29]. Exudates from dermal lesions were spotted onto microscope slides, inoculated into a culture medium, and processed for DNA extraction. Blood samples from two children with symptoms of visceral leishmaniasis and 18 members of their family and a dog living in the same house were sampled in an EDTA coated flask (Vacutest Kima, Arzergrand, Italy).

### 2.5. Parasitological Diagnosis

Direct microscopic examination of smears was performed for all suspected CL patients. Thus, smears were stained with Giemsa and examined at 100× magnification. Exudates were seeded in an NNN medium supplemented with few drops of antibiotic (Penicillin 1 U/mL) in sterile saline solution [26,27] and then sub-cultured every week for four weeks. The buffy coat extracted from the blood of VL cases, their family members, and the dog samples were inoculated into both NNN and rabbit’s coagulated serum medium [30].

### 2.6. Molecular Diagnostic and Typing

DNA was extracted from the exudates of lesions of 96 patients suspected of CL as well as from the previous 21 blood samples using the QiAamp DNA Blood Mini Kit (Qiagen^®^, Hilden, Germany), according to the manufacturer’s instructions. The DNA was eluted in 50 µL of Tris EDTA (TE) buffer and stored at −20 °C until used. Promastigotes of the following three *Leishmania* reference strains were used as a control: *L. infantum* (MHOM/FR/78/LEM75), *L. tropica* (MHOM/DZ/05/LIPA07/05), and *L. major* (MHOM/DZ/82/LIPA80).

Typing was performed by PCR-ITS1 (Polymerase Chain Reaction-Internal Transcribed Spacer 1 genes) and RFLP (Restriction Fragment Length Polymorphism). The amplification was carried out in a volume of 50 μL containing 5 μL of DNA, 4.0 mM of MgCl_2_, 200 μM dNTP’s, 500 nM of each primer (LITSR: 5′-CTGGATCATTTTCCGATG-3′, and L5.8S: 5′-TGATACCACTTATCGCACTTA-3′), 2 U of Taq polymerase. The amplification conditions were 94 °C for 4 min, followed by 36 cycles of 95 °C for 40 s, 53 °C for 30 s, 72 °C for 60 s, and 72 °C for 6 min [28]. DNAs of reference strains and water were used as positive and negative controls. The amplification product was analyzed on a 2% agarose gel and visualized under ultraviolet light. The presence of the 300 and 350 base pair bands compatible with those of the reference strains was analyzed. Then, positive samples were incubated at 37 °C for 24 h with the endonuclease enzyme HaeIII (Hybaid GmbH, Heidelberg, Germany) to identify the parasite at the species level. After electrophoresis on 2% agarose gel and visualization under ultraviolet light, the lengths of the bands obtained were compared with those of the reference strains [28].

Real-time qPCR amplification of the kDNA was performed were carried out in a volume of 25 μL containing 1 µL of DNA in 24 µL of a master mix (Taq Man Universal Master mix II with UNG (Applied)) having 100 nM of each primer (5′-CTTTTCTGGTCCTCCGGGTAGG-3’) and (5’-CCACCCGGCCCTATTTTACACCAA-3′), 50 nM the probe (FAM 5′-TTTTCGCAGAACGCCCCTACCCGC-3′). The DNA was amplified with a Taq Man Applied Biosystems 1 apparatus (Applied Biosystems, Foster City, CA, USA). The amplification conditions were 95 °C for 2 min, followed by 40 cycles of 95 °C for 15 s, 60 °C for 1 min, and 60 °C for 1 min. Reactions were validated with positive and negative control samples. The DNA of the samples was quantified against a serial dilution of *Leishmania* DNA extracted from a known number of parasites (0.5 to 0.005 parasites per reaction tube). Real-Time PCR was considered positive when the test sample’s cycle threshold was below that of the last point of the range, in a valid reaction [31].

### 2.7. Sequencing and Phylogeny of Chosen Isolates

ITS1 amplicons were purified using ExoSAP (ThermoScientific, EU). *Leishmania* species were identified by DNA sequencing using an ABI Prism1 Big Dye™ Terminator, Cycle Sequencing Ready Reaction Kit, and AB1 3130 sequencing system (ABI, PE Applied Biosystems), with the same primers used for PCR. DNA sequences from both strands were aligned and edited using the Staden software package [32]. The MEGA version 7 software [33] was used to conduct multiple sequence alignments (ClustalW option) and construct a phylogenic tree. Homology searches were performed using BLAST (Basic Local Alignment Search Tool) using the NCBI (National Center for Biotechnology Information) server [34]. The generated sequences were aligned using SeaView4 [35] to study the targeted genes’ genetic variability. Phylogenetic analysis was carried out using maximum likelihood analysis with the online phylogeny website (www.phylogeny.fr, accessed on 15 October 2020) [20,36]. Alignments were done with the ITS1 sequences of *L. donovani* from Senegal (MN244151.1) and China (MH202978.1), *L. infantum* from Italy (KU680955.1), Morocco (KX664454.1) and France (AJ634339.1); *L. tropica* from Morocco (KM454152.1), Libya (KP691595), Tunisia (JN104588.1); *L. aethiopica* from Ethiopia (FN677356.1); *L. major* from Iraq (KY882275.1), Uzbekistan (FN677357.1), Tunisia (FN677342.1); *L. panamensis* (HG512959.1); *L. braziliensis* (HG512942.1); *L. martiniquensis* (MG731229.1). *C. fasciculata* was used as an outgroup (JX683017.1).

### 2.8. Multilocus Enzymatic Electrophoresis (MLEE) Typing

Eighty-seven isolates were analyzed with MLEE (66 from humans and 21 from rodents, including 23 isolates collected during the survey performed in 2018/2019. The remaining 43 human isolates were taken from the cryobank of the Institut Pasteur of Algeria. The isoenzyme characterization of isolates was carried out using 15 enzyme systems, according to the protocol previously described by Rioux et al. [37].

### 2.9. Data Management and Statistical Analysis

Data management and analysis were performed using Excel 365 (Microsoft office, USA); Graph-prism (8.02, GraphPad Software Inc., San Diego, CA, USA); XL STAT (v2020, Addinosoft) and Stata 11 (v16.1, StataCorp, TX, USA). Maps that relate the spatial distribution of CL cases and the cumulative incidence within the 48 provinces and the cumulative number of patients in the 15 districts of M’Sila were constructed using Photoshop (v2015, Adobe). A one-factor ANOVA analysis used to test CL per province’s incidences per year was performed using the Graph-prism (v8.02, GraphPad Software Inc). and Stata 11 software. Dunnett’s post-test was used when the ANOVA *p*-value was less than 0.05. A principal component analysis (PCA) was performed using XL STAT.

### 2.10. Ethical Considerations

The study protocol, informed consent forms, and sampling/experimental procedure were reviewed and approved by the Ethical Committee of Institut Pasteur of Algeria (the study began after obtaining the informed consent from patients under Helsinki’s declaration (1964)). All animal experiments carried out in vivo comply with the guidelines of the Veterinary Inspection department of Algiers (veterinary health declaration established by the chief veterinary inspector, N ° 2505 / IVWA / 2019 in April 2020 and approved by the Research Ethics Committee of the Institut Pasteur of Algeria, and also approved by the Algerian Association for the Sciences of Animal Experimentation (AAAES).

## 3. Results

### 3.1. Dynamics of Cutaneous Leishmaniasis in the Province of M’Sila Compared to the National Epidemiological Status of the Disease

**Figure 4:**
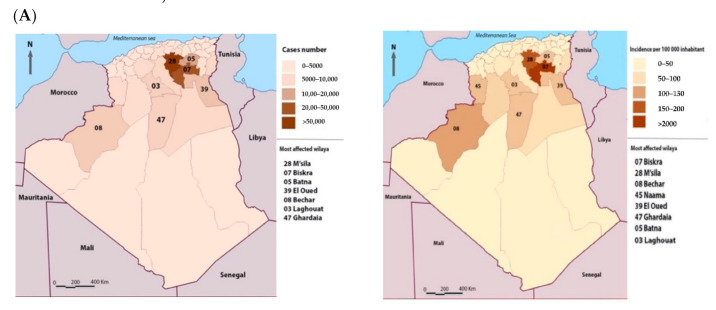

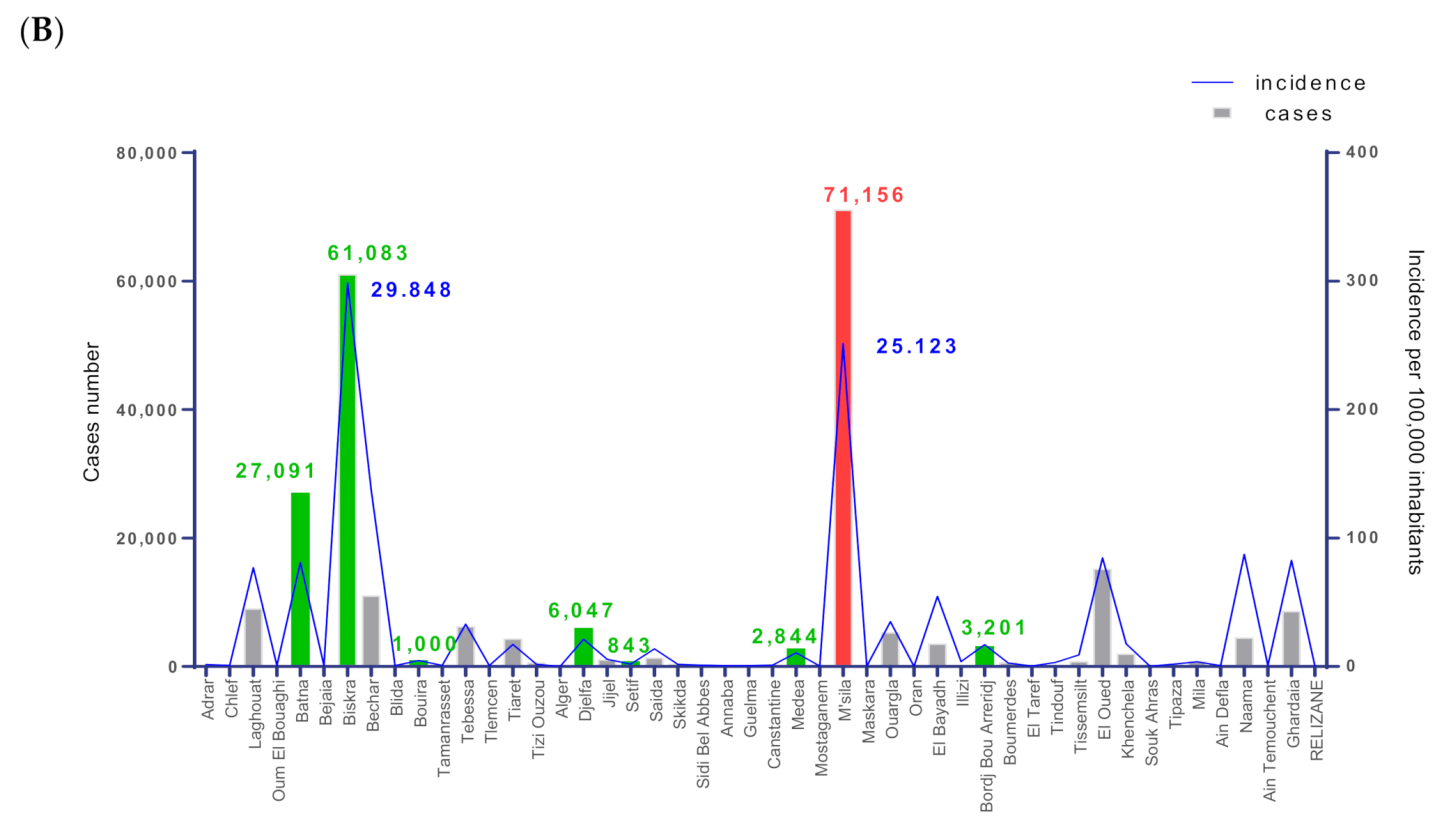
Total case number and incidence recorded during the 1982 to 2017 period. (**A**) The geographic location of registered cases (**right panel**) and incidence (**left panel**) during the 1982 to 2017 period. (**B**) Total cumulative cases and incidences per province during the period from 1982 to 2017. Provinces bordering M’Sila (red) are denoted by green color.

From 1982 to 2017 (34-year period), more than a quarter of a million cases (252,659) of CL were registered in the country (Figure 4A). During this period, five provinces (Bechar, El Oued, Batna, Biskra, and M’Sila) recorded more than 70% of the total cases (Figure 4B).

**Figure 5:**
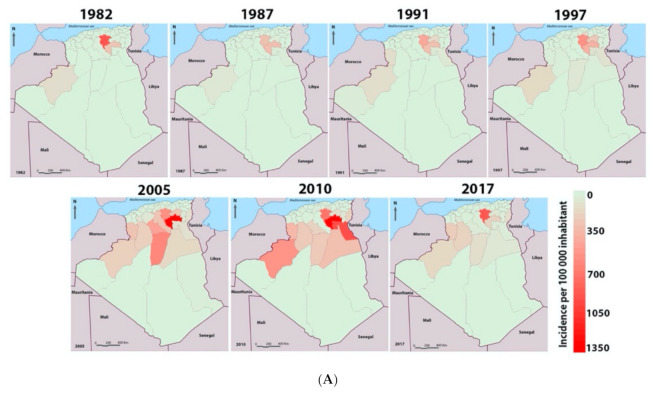

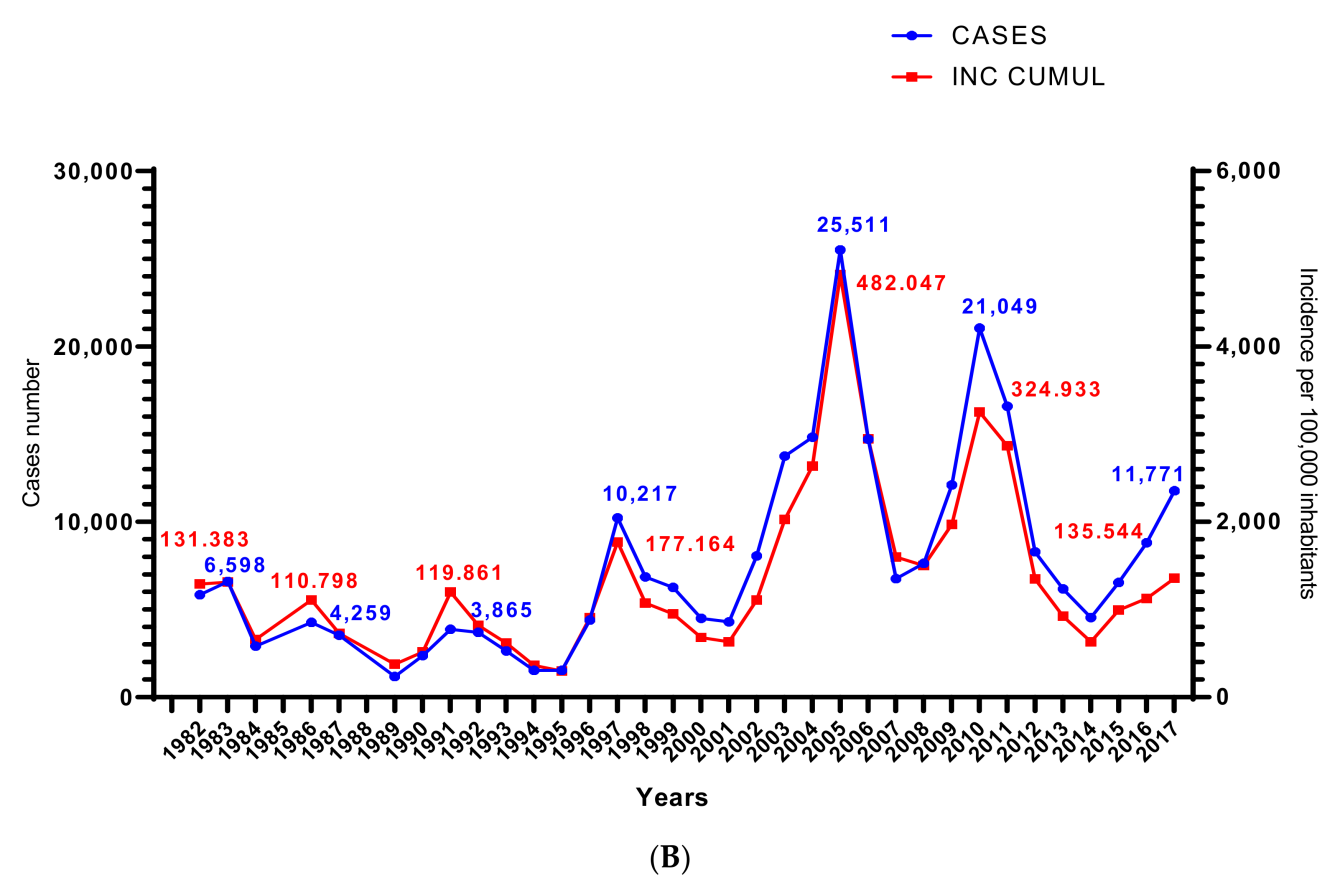
Total case number and incidence of cutaneous leishmaniasis from 1982 to 2017 in Algeria. (**A**) Spatial distribution of cutaneous leishmaniasis cases during the seven epidemic outbreaks recorded in Algeria. (**B**) Cutaneous leishmaniasis cases cumulative incidence (INC CUMUL) in Algeria over the period from 1982 to 2017.

Since 1982, the dynamics of CL reveals an endemic-epidemicity status of the disease, with several peaks observed, the most critical being in 2005 with more than 25,511 cases recorded in the country (Figure 5B). Statistical analysis disclosed a highly significant difference (ANOVA 1-way test, *p* < 0.0001) in the annual distribution of recorded cases over the 34 years. As compared to 2005, the incidence appears to be significantly different to other years, except for 2004, 2006, 2010, and 2011. The disease’s endemic-epidemicity is also observed in M’Sila, a province with approximately 90,000 cumulative cases since 1982 and seven epidemic peaks in 1982, 1987, 1991 and 1997, 2005, 2010, and 2017 (Figure 5A,B).

**Figure 6:**
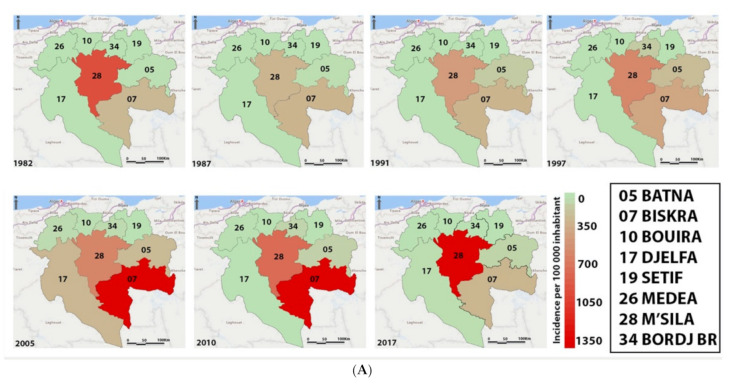

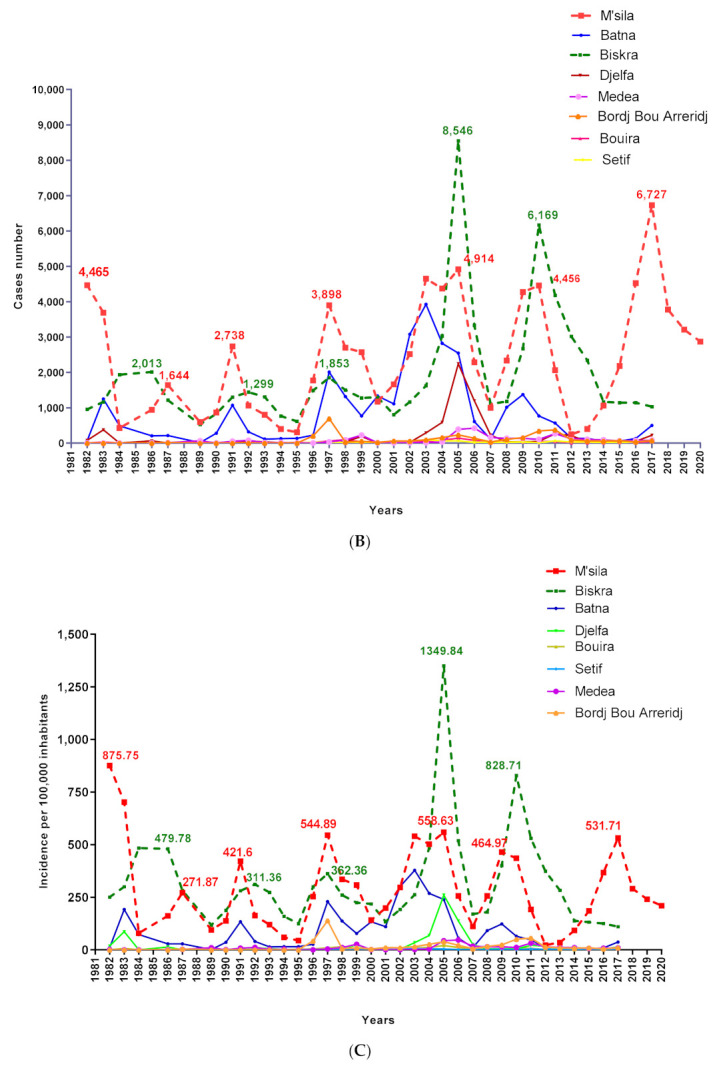
Representation of cutaneous leishmaniasis incidence in M’Sila and the seven bordering provinces. (**A**) Spatial representation of CL incidence during the seven major epidemic outbreaks. (**B**) Cases number and (**C**) incidence of cutaneous leishmaniasis during the period from 1982 to 2020.

The district of M’Sila is significantly more affected in terms of the number of cumulative cases than the rest of the districts (1-way ANOVA test, *p* < 0.0001). A higher CL case incidence was reported in M’Sila than in the former foci of Biskra (Dunnett’s post, *p* < 0.05) (Figure 6A,B).

**Figure 7:**
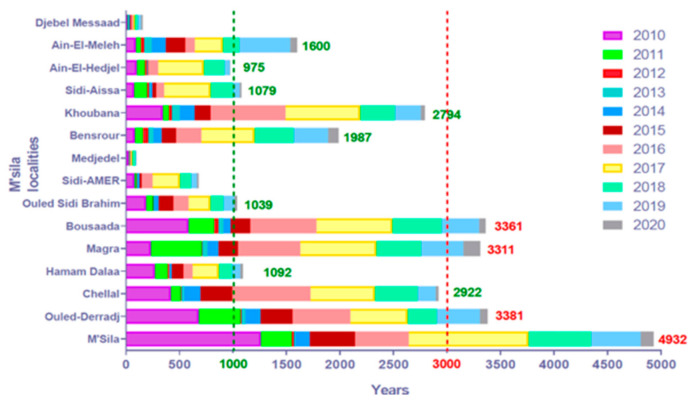
Temporal dynamic of cutaneous leishmaniases cases recorded in the 15 districts of the province of M’Sila over the last decade (2010–2020).

The analysis of CL cases registered in the province of M’Sila from 2010 to 2020 depicts that all districts are affected. Four out of the 15 districts reported more than 3000 cumulative cases during the last decade, M’Sila (4932), Ouled Derradj (3381), Boussaâda (3361), and Magra (3311), respectively, followed by the district of Chellal, Hammam Dalaa, Bensrour, Khoubana, Ain El Melh, and Sidi Aissa which recorded less than 3000 cases (Figure 7).

### 3.2. Cutaneous Leishmaniasis in the M’Sila Province

#### 3.2.1. Lesion Characteristics, Patient Stratification, and Environmental Features

Of the 96 suspected CL patients, 74% [65.4–82.6] were positive for at least one of the used diagnostic methods (i.e., direct microscopic examination, in vitro culture, PCR-ITS1, qPCR). Fifty patients (71.6% [59.7–82.1]) presented ulcerative lesions and 13 patients a nodular form (19.4% [10.4–29.9]). Forty-three patients (61.4% [50.0–72.8]) had more than one lesion, among whom 11 (15.7% [7.1–24.3]) exhibited a minimum of 4 lesions. Arms and legs are the most affected parts of the body (50 patients, 70.4% [59.6–81.2]). A single facial lesion was observed in 21 patients (29.6% [18.8–40.4]). Patient age: CL affected all age categories from 9 months to 73 years, with 7.5 years as a median age. Fifty-four patients (76.1%) were less than 15 years old, with the most affected less than 5 years old (31 cases, 43.7% [32.4–54.9]), followed by those between 6 and 10 years of age (15 cases, 21.1% [11.3–31]). Patients over 15 years of age represented 23.9% of the total cases. Females are slightly more affected than males (40 females: (56.3% [45.1–67.6], 31 males: (43.7% [32.4–54.9]), with a sex ratio of 1.3. The sex ratio was equal to 1 in children under 5 years of age. The highest number of cases was recorded in the Daïras of Boussaâda (18 cases, 25.4% [15.5–36.6]) and M’Sila (14 cases, 19.7% [11.3–29.6]). Most patients live in rural areas, 66.2% [54.4–76.5]. The presence of domestic animals or rodents in or near a dwelling was reported by 41 patients (59.2% [47.9–70.4]). Thirty-seven patients (52.1% [43.9–68.2]) signaled CL cases among their family (85.3%) or in their neighbors (20.6%).

#### 3.2.2. Leishmania Detection and Identification in Patients and Reservoirs

The results of the diagnostic methods performed on skin samples of humans suspected of CL are summarized in Table 3.

**Figure 8:**
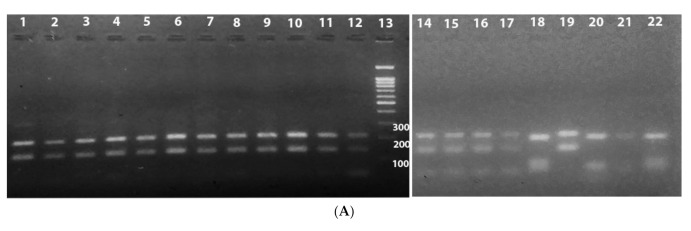

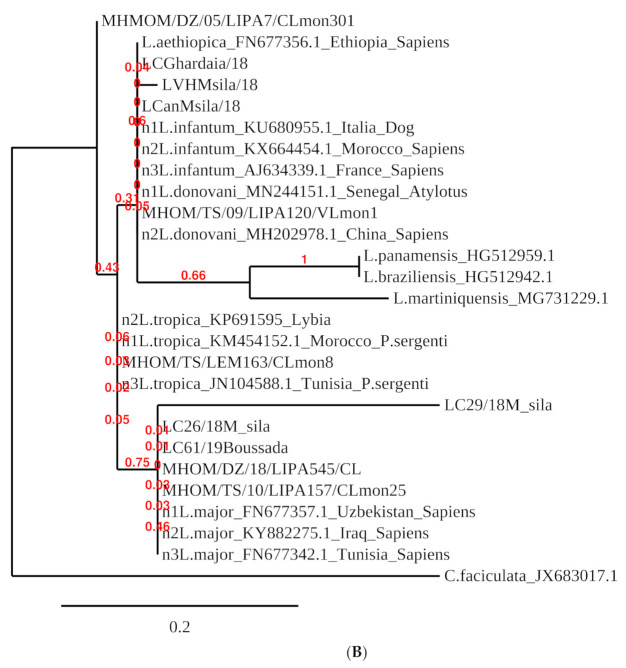
Identification of *Leishmania* isolates using molecular methodologies. (**A**) PCR-RFLP of ITS1 as revealed after agarose gel electrophoresis. Lane 1, reference strain LIPA80 (*L. major* Mon-25); lane 2 to 9, isolates from Human Cutaneous Leishmaniasis; lane 10 to 11, isolates from *P. obesus*; lane 12, isolate from *M. shawi*; lane 13, MW (molecular weight marker); lane 14, reference strain LIPA80 (*L. major*); lane 15, a suspected variant of *L. major* strain (LIPA545/17); lane 16, LC61 (Leishmania isolate from Boussaâda); lane 17, LC80 (Leishmania isolate from Boussaâda); lane 18, reference strain LEM75 (*L. infantum* Mon-1); lane 19, LC61 (see above); lane 20, references strain LIPA07/05 (*L. tropica* Mon-301); lane 21, isolates from canine leishmaniasis (*L. infantum*); lane 22, isolates from human visceral leishmaniasis cases (*L. infantum*). (**B**) Phylogenetic reconstruction from ITS1 sequences of *Leishmania* isolates and references strains. An amplified fragment of the ITS1 gene was sequenced from 7 isolates (1 VL, 1 CanL, and 5 CL). Reference strains are *L. infantum* MHOM/FR/78/LEM75, *L. killicki* (*syn L. tropica*) MHOM/TN/80/LEM163, and *L. major* MHOM/MA/81/LEM265 (7).

Among the suspected CL patients, 45.83% were recorded positive based on direct examination and 53.68% based on culture. A higher positivity rate was recorded for molecular methods, reaching 73.40% using qPCR. In total, 71 patients were diagnosed as CL positive (Table 3). Molecular identification (PCR-RFLP) disclosed that all 42 CL samples and 21 gerbil samples tested were positive for *L. major*. Samples collected from humans with visceral symptoms (two cases) and from a dog (one case) belong to *L. infantum* (see Figure 8). Isolates characterized by MLEE (66 from humans and 21 from rodents) were identified as *L. major* zymodeme MON-25. A minor variation in 3 enzymatic complexes for G6PD, PGD, and ME is recorded in 10 isolates, suggesting the possible existence of *L. major* variants in this region. No discrepancy was observed between PCR-RFLP and MLEE results. A fragment amplified from the ITS1 gene was sequenced for seven isolates (1 VL, 1 canine leishmaniasis, and 5 CL). Reference strains used were *L. infantum* MHOM/FR/78/LEM75, *L. killicki* (*syn L. tropica*) MHOM/TN/80/LEM163, and *L. major* MHOM/MA/81/LEM265 (7). *L. infantum* was identified in visceral leishmaniasis and canine leishmaniasis cases, whereas *L. major* was identified in CL cases. The identification of *L. major* in lesions of CL and the presence of *L. infantum* in dog and human suspected cases of VL were confirmed by the phylogenetic analysis of the ITS1 sequences (Figure 8B).

### 3.3. Entomological Survey in the Province of M’Sila

#### 3.3.1. Diversity and Dynamic of Sandfly Populations

A total of 10,706 specimens were caught, 99% of which were collected during the 2003/2004 season and only 79 in 2018. Morphological identification revealed 16 species belonging to the genus *Phlebotomus* (89.53%) and *Sergentomyia* (10.47%). Details are given in Table 4 and Table 5. Six new species have been identified for the first time in the province of M’Sila: *P. perfiliewi*, *P. langeroni*, *P. chabaudi*, *S. clydei*, *S. schwetzi*, and *S. lewisi*.

**Figure 9:**
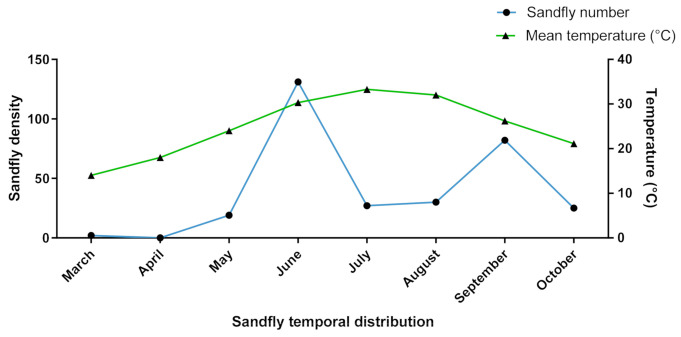
Seasonal dynamics of P. *papatasi* as recorded during the 2003/2004 transmission period.

Within the M’Sila province, *P. papatasi* is highly prevalent, particularly in the districts with high CL incidence, *P. longicuspis* being abundant in districts where few cases of VL are recorded. The sandfly dynamics recorded in the province of M’Sila highlight a relation between sandfly density and the average temperature recorded. The sandfly population density reaches its first peak in June and a second in September (see Figure 9).

In Boussaâda, 12 species were identified; *P. papatasi* is highly abundant in this station (74.83%), followed by *P. longicuspis* (18.65%). Two species were detected for the first time in this region, *P. chabaudi* (4.29%) and *S. lewisi* (0.06%). In the district of M’Sila, the specimens collected belonged to 12 species. The Phlebotomus genus represents 86.78% of the total sample, and *P. longicuspis* (53.25%) was the most prevalent. Notably, *P. papatasi* represented 33.84% of the specimens captured, and *S. clydei* was identified for the first time in this region. In Chellal, specimens belonging to 10 sandfly species were recorded. *Sergentomyia* were prevalent and represented 71.38%, including *S. fallax* (46.53%), *S. antennata* (38.79%), *S. schwetzi* (8.78%), and *S. minuta* (5.77%). *Phlebotomus papatasi* represented 27.12% of sandflies captured in rodent burrows. In this district, *P. perniciosus* (3%), *P. longicuspis* (1.31%), *P. alexandri* and *P. chabaudi* (0.65%) were also recorded. In Magra, 6 species were identified, with *P. papatasi* (53.85%), *P. perniciosus* (19.23%), and *P. longicuspis* (6.73%) being the most frequently trapped. At Ain El Hadjel, 10 species were identified. *Phlebotomus papatasi* is the most abundant (91%), followed by *P. perniciosus* (6%). Four specimens of *P. perfiliewi* were collected for the first time in this arid region. A total of 7 species were identified at Ouled Derradj. *Phlebotomus perniciosus* is the most frequently identified (66%), followed by *P. sergenti* (11.96%), *P. papatasi*, and *P. longicuspis* (both < 10%). In the districts of Sidi Aissa, Ain El Melh, and Hammam Delaa, a low number of sandflies were collected, with a large majority represented by *P. papatasi* (78%).

#### 3.3.2. Biotope and Distribution of Sandflies in the Province of M’Sila

**Figure 10:**
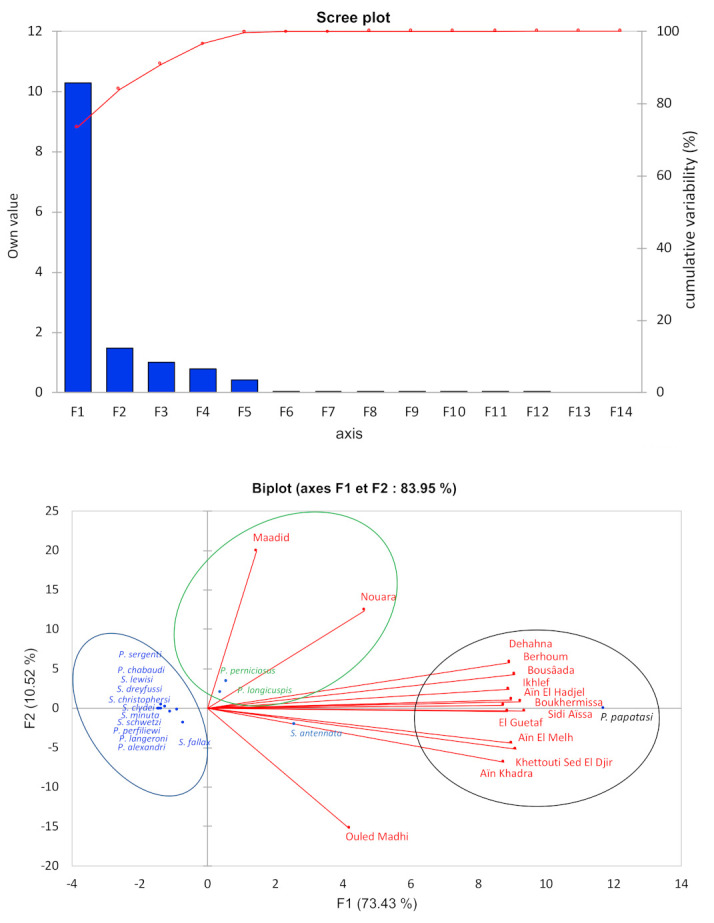
PCA analysis of sandfly species abundance in the districts belonging to the M’Sila province, according to the biotopes. Upper panel: calculated eigen values, Lower panel: representation of the biplot (F1 and F2 axes) analysis.

*Phlebotomus papatasi* represents a non-negligible proportion of sandflies collected in the province of M’Sila (65.93%). Indeed, this species is predominant in the stations of Ain El Hadjel (91%), Sidi Aissa (78%), Boussaâda and Ait Ikhlef (75%), Berhoum (69.70%), Ain El Melh (65%), Khettouti Sed El Djir (63.41%), Ain El Khadra (54.55%), Dehahna (45%), El Guetaf (43.75%), Boukhmissa (42.2%). *P. papatasi* is less frequent in Nouara (27.6%), Ouled Madhi (19.6%), and Ouled Derradj (9.3%). On the other hand, *P. longicuspis* was relatively abundant in two stations; the garden areas with apricot trees in Nouara (51.82%) and the palm grove of Boussaâda (19%), which both are irrigated. *Phlebotomus perniciosus* is abundant in Ouled Derradj (66.11%), Dehahna (23.33%), and Berhoum (18.18%). These three species represent 86.48% of all specimens collected. Two axes of the principal component analysis were retained (F1 and F2) since their calculated eigenvalues are close to 1 (Figure 10, upper panel).

The factorial plane displays that *P. papatasi* is the main contributor of the F1 inertia, and its abundance correlates with arid steppe, plains, and foothills, and less frequently with mountains (9.3%). Steppes offer optimal ecological conditions for this species [7,24,38,39]. *Phlebotomus perniciosus* and *P. longicuspis* are the main contributors to the axis 2 inertia. A third group encompasses other sandfly species that, for the majority, are not of medical importance (Figure 10, lower panel).

### 3.4. Survey of the Reservoir Hosts of Cutaneous Leishmaniasis

More than 445 wild rodents belonging to 5 families, 7 genera, and 9 species were collected during the study (Table 2). Gerbils are abundant in the plains and the Chott (Table 2). *Ctenodactylus gundi* (Ctenodactylidae) is frequent in the Rocky Mountains of Boussaâda and Ain Farez (Daïra of Ain el melh), located 30 km south-east of Boussaâda city. *Psammomys obesus* and *Meriones shawi* often overlap in terms of territories. *Psammomys* *obesus* was trapped frequently in the steppe where Chenopodiaceae develop, the Chellal region, and the road shoulders along the Sebkha (around the Chott). This rodent species was also trapped at the piedmont of Jebel Choukchot’s mountains near Sidi Aissa and in peripheral areas of M’Sila. Like *M. shawi*, it settled on the slopes at the edge of cereal fields. It is also abundant in orchards and agricultural areas. It was captured near the salt-lake (Sebkha) of M’Sila, in the countryside and its suburbs. Molecular diagnosis by PCR-RFLP revealed the infection by *Leishmania* in only two species, *P. obesus*, and *M. shawi*. Among the 186 *P. obesus* and the 37 *M. shawi* collected during the survey, 29.57% and 27% were positive for *Leishmania* DNA, respectively (Table 6). The 21 isolates of *Leishmania* from rodents (*P.* *obesus* ₌ 17, *M. shawi* ₌ 4) were identified as *L. major* MON-25 (see above).

## 4. Discussion

### 4.1. Epidemiology of Leishmaniases in Algeria and M’Sila 

In Algeria, the first case of human CL was described in 1860 by Hamel in the former focus of Biskra [9] and 1924 in M’Sila [10]. The disease evolved slowly until the early 1980s when outbreaks occurred suddenly in M’Sila, which is very close to Biskra. Since then, the disease has become a real public health problem, and its incidence has increased significantly in the recent decades [4,40]. After 34 years (1982–2017), more than a quarter of a million cases were recorded in all the country (252,659 cases), which equals an incidence of 1372 cases/100,000 inhabitants. Five provinces are highly endemic for CL, with more than 10,000 cases recorded between 1982 and 2017. Three are located in the west of the country and are adjacent to Biskra (Batna, M’Sila, and El Oued). M’Sila and its seven neighboring provinces account for 74.5% of the total CL cases reported in Algeria during the 34 years of our study. M’Sila and Biskra alone account for 37.22% of the recorded cases during these three decades. A Dunnett’s statistical analysis discloses no significant differences in terms of incidence between these two provinces. The temporal dynamics of the CL case number follow that of M’Sila, showing the endemic-epidemic profile of the disease with a high proportion of cases in the direct neighborhood of patients (56.1%).

### 4.2. Leishmania Species and Clinical Presentation in M’Sila 

Parasite identifications carried out during our prospective and retrospective surveys (87 by MLEE, 42 by PCR-RFLP) confirmed *L. major* as the etiological agent of CL in the M’Sila region and are in accordance with previous studies [3,12,41]. The most common zymodem in Algeria (MON-25) [12,41] was the sole identified. However, variability was recorded for three enzymatic systems, G6PD, PGM, and ME, in some isolates from Boussaâda, Sidi Aissa, and M’Sila. *Leishmania major* MON-269 as a causative agent of CL was previously reported in Algeria [41]. This zymodem differs from MON-25 by a single enzyme system (PGD 122/94) and could correspond to a recent mutation or a post-translational modification [42]. The combination of four diagnostic methods (microscopy, NNN culture, ITS1-PCR, and RT-PCR) allowed diagnosis confirmation of a maximum number of cases among the suspected CL patients, resulting in a more accurate clinical description of the disease. As reported [31,43], RT-PCR was the most sensitive (100%) technique. On the other hand, the high sensitivity of cultures (72.9%) is related to the adaptation of *L. major* to the NNN medium [27]. The clinical appearance is characterized by a predominance of childhood infection and multiple lesions (61.4%), preferentially localized to the limbs (70.4%) [44]. Children of less than or equal to 15 years (76.1% of cases) considered as “naive” are likely to be the most susceptible to *L. major* infection [45,46].

Otherwise, M’Sila does not fall into the group of provinces where VL is endemic; therefore, the identification of *L. infantum* in dogs and humans requires further studies to delineate the endemic occurrence of the transmission cycle.

### 4.3. Sandfly Diversity and Dynamics in Algeria and M’Sila 

Out of the 24 species reported in Algeria [24,47,48], 16 were identified during this study, mostly in sympatry in the various districts investigated. The comparable richness in sandfly fauna was reported in the north/north-eastern and middle eastern regions of Algeria [49,50,51]. Our study enriched the sandfly entomofauna of M’Sila [52] with 6 new species, *P. chabaudi*, *P. langeroni*, *P. perfiliewi*, *S. clydei*, *S. schwetzi*, and *S. lewisi*. The high proportion (65.93%) of *P. papatasi* in surveys performed during 2003–2004 and 2018 and reported by Boudrissa in M’Sila and the neighboring provinces [4] indicates that sandfly richness might not have changed over time in this area. Likewise, *P. papatasi* being the proven vector of *L. major*, zoonotic CL incidence, is high in this area [15,53].

Interestingly, some identified sandfly species with high densities in some districts, such as *P. perniciosus* and *P. longicuspis*, are suspected or proven vectors for visceral leishmaniasis. The sandfly seasonal dynamics revealed a bell-shaped density curve with a broad peak encompassing the July–September period and between early October to late May. The season for leishmaniasis transmission risk took place from early June through the end of September when maximal density is achieved. This kind of bell-shaped density curve was described for sandflies in the Mediterranean area [54]. During this period, the second and third generations of sandflies are more abundant, increasing the infection risk [53]. The ZCL seasonal dynamic is under minimal temperature control and presents a 2-month lag between the reported infection date and the presumed date of CL lesion onset [55,56].

### 4.4. Ecology, Diversity, and Medical Importance of Sandflies in M’Sila 

The 5 stations analyzed with PCA revealed a predominance of *P. papatasi* in stations above 300 m of elevation, plains, and piedmonts and coincided with the ZCL foci of M’Sila. In these areas, steppes constitute the optimal ecological niche for *P. papatasi*. *Phlebotomus papatasi* can also be present at a higher altitude, up to 1000 m, like in Maadid, where it accounted for 9.30% of the total specimen trapped. In lowland rural areas, *P. papatasi* reach up to 91% (Ain El Hadjel, Sidi Aissa, and Boussaâda). The low sanitation level would favor the presence of manure and organic matter that, along with high humidity, are adequate for sandfly breeding [57]. *Phlebotomus perniciosus* and *P. longicuspis* are abundant mainly in the piedmont area of Nouara (M’Sila) and the mountain of Maadid (Ouled derradj), respectively. *Phlebotomus longicuspis* is abundant in humid peri-urban regions, like in Nouara, or Boussaâda palm grove, representing 51.82% and 18.65% of the total trapped sandflies, respectively. It is a suspected *L. infantum* vector in the arid zones of Morocco and Tunisia [58,59]. *Leishmania infantum* has been detected in *P. longicuspis* in Algeria [60]. During our survey in 2018, two autochthonous VL cases and one canine case were detected in Maarif (a few kilometers from Boussaâda city). However, the potential vectorial role of *P. longiscupis* for *L. infantum* requires to be proved more thoroughly in M’Sila. Only seven specimens of *P. perfiliewi*, a proven vector of CL caused by *L. infantum* in Algeria [61,62] and Tunisia [63], and of VL in Algeria [64], were captured during our survey. Common in the humid and subhumid areas, this sandfly was collected in the highlands’ arid region, where conditions might not be optimal for this species [24]. This species is abundant in the semi-arid bioclimatic stages, like in Constantine [50,65]. Specimen of the paraphlebotomus genus were less frequently trapped. *Phlebotomus sergenti* represented 0.68% of the total specimen, but it reached up to 12% in the Maadid station in Ouled Derradj. In Algeria, this species is mainly encountered in the south of the country in the Saharan Atlas’ foothills, the pre-Saharan steppes, and the Tell regions [24]. It was found in crevices of walls, rodent burrows, and rocky areas [24]. It is the proven vector of *L. tropica* [66] in the Middle East and probably of *L. killicki* (syn: *L. tropica*) in the Maghreb countries [67]. *Leishmania killicki* has been identified in many CL cases in Ghardaia in the center of Algeria [68]. *Phlebotomus chabaudi* was observed in the Boussaâda palm grove, and the Chellal region (Ouled Madhi). Since its description in Tunisia [23], the presence of this species is documented in Ghardaïa in Algeria [69] and Morocco [70]. The presence of *P. chabaudi* in the biotopes of *Ctenodactylus gundi* (rocky biotopes) would make them a potential vector of *L. tropica*. *Phlebotomus (Par). Alexandri*, collected in the district of Ain El Hadjel, is considered as a ZCL vector in the former USSR and Iran [71,72]. Also, in this district a single specimen of *Phlebotomus (Lar) langeroni* was collected. It is considered as a vector of *L. infantum*, responsible for sporadic CL in Northern Tunisia [73] and of VL in Egypt [74,75]. It is particularly abundant in rocky and dry biotopes where *Ctenodactylus gundi* and reptiles roost [24].

### 4.5. Rodents and Reservoirs in the M’Sila Province 

In Algeria, the endemicity of the ZCL is linked to a regular epidemiological cycle maintained by *P. obesus*, whose populations are geographically restricted. In contrast, epidemic outbreaks correlate with the proliferation of *M. shawi* and food abundance during rainy years. The surveys have made possible an update of mammalian fauna inventory and a more accurate spatial and micro-zonal distribution of rodents playing a role in ZCL transmission. Several ecological niches were identified, and nine rodent species were inventoried. This number does not reflect the rodent fauna richness in this region since the inventory performed in 1991 by Kowalski and Rzebik-Kowalska [76] reported 19 rodent species in the Hodna basin. During our survey, only *P. obesus* and *M. shawi* were found infected by *L. major* MON-25 (Table 2). The presence of *L. major* in *M. shawi* trapped in Sidi Aissa (1996), and Ain El Khadra (2000) (Table 2) represent the first report of such events in the M’Sila province. The molecular diagnosis detected *L. major* in 29.57% of *P. obesus* and 27% of *M. shawi* trapped. This confirms the primary reservoir’s role of these two species in the province of M’Sila.

Interestingly, in a previous study, a Leishmania isolates from *Meriones* spp. was categorized as non-susceptible (S−) toward trivalent and pentavalent antimony [6,77]. Further experiments are required to address the risk of chemotherapeutic failure in link with the transmission of such variants.

## 5. Conclusions

Zoonotic CL caused by *L. major* is highly prevalent in the M’Sila region for more than 30 years, with an average of around 3000 cases per year. This focus is characterized by an arid bioclimate, a high density of *P. papatasi* sandflies, and a high *Leishmania* infection rate of *Psammomys obesus* and *Meriones shawi* rodents. The clinical profile of the disease is similar to that described in other North African countries, CL being most frequently encountered mainly in childhood and presenting as multiple lesions preferentially localized on the limbs. Identifying ZCL risk factors in M’Sila could allow more efficient control of the disease.

## Figures and Tables

**Table 1:** Description of stations sampled during the entomological survey performed in 2003/2004 and 2018.

Districts	Station Name	Geo Reference	Elevation (m)	Description	Specimen	
					n	%
M‘sila	Naoura	35°39′ N/04°31′ E	475	Rural area. Ovine and bovine population.	1264	11.81
	Boukhmissa	35°43′ N/04°33′ E	475	Urban area, with a bovine population.	173	1.62
Chellal	Ouled Madhi	35°34′ N/4°30′ E	415	Rural area. South of M’Sila, outskirts of Hodna’s chott. Presence of rodent burrows.	908	8.48
	Khettouti Sed El Djir	35°37′ N/4°10′ E	460	Rural area.	82	0.77
	Ait Ikhlef	35°36′ N,4°29′ E		Rural area.	79	0.74
Magra	Berrhoum	35°39′ N/5°2′ E	596	Rural area.	33	0.31
	Ain El Khadra	35°32′ N/4°58′ E	446	Rural area.	11	0.10
	Dehahna	ND	ND	Rural area.	60	0.56
Hammam Dalaa	El Guetaf	ND	ND	ND	64	0.60
OuledDerradj	Maadid	35°55′ N/04°22′ E	1000	Rural and semi-arid areas.	301	2.83
Ain El Hadjel	Ain El Hadjel	35°40′ N/03°52′ E	500	Steppe with farming	2773	25.90
Sidi Aissa	Sidi Aissa	36°30′ N/04°17′ E	1003	Rural area.	50	0.47
Ain El Melh	Ain El Melh	34°50′ N/04°9′ E	948	Rural area.	20	0.19
Bou Saâda	Boussaâda	35°12′ N-04°12′ E	ND	Palm grove and Oued. Presence of goats, chickens, and pigeons	4888	45.66

ND: not determined, n: number of samples collected, %: percentage compared to the total number of catches done.

**Table 2:** Description of stations sampled during the survey of rodents performed from 1996 to 2018.

Locality	Family	Genus/Species	Year	N Tot	N Inv	N Inf	N Isol
Chellal, Sidi Aissa, other	*Gerbillidae*	*Psammomys obesus*	1996	ND	ND	ND	3
			2003	ND	10	7	4
			2004	60	30	5	4
			2007	ND	ND	ND	6
			2010	223	144	43	-
			2018	ND	12	-	-
M’Sila, Sidi Aissa, Magra, Ain-El-Hadjel	*Gerbillidae*	*Meriones shawi*	1996	ND	ND	ND	1
			2000	ND	ND	ND	1
			2004	ND	10	3	-
			2008	49	20	6	-
			2009	ND	ND	ND	2
			2012	12	6	1	-
			2018	ND	1	-	-
M’Silasila	*Gerbillidae*	*Meriones lybicus*	ND	13	6	-	-
Boussaâda, M’Sila, Ain-El-Hadjel, Khoubana	*Gerbillidae*	*Gerbillus sp.*	ND	46	ND	-	-
			2018	ND	3	-	-
Boussaâda	*Gerbillidae*	*Ctenodactylus gundi*	ND	12	12	-	-
Ain-El-Melh			2018	ND	2	-	-
M’Sila, Hamama, Delaa	*Dipodidae*	*Jaculus jaculus*	ND	12	6	-	-
Ain-El-Melh, Khettouti, Sed-El-Djir	*Dipodidae*	*Jaculus orientalis*	ND	11	4	-	-
Ain-El-Mehl, Boussaâda, Hamama, Dalaa, Khettouti, Sed-El-Djir	*Muridae*	*Rattus ratus*	ND	5	5	-	-
Hamama, Dalaa	*Macroscelidae*	*Elephantus rozeti*	ND	2	2	-	-
			Total	445	273	65	21

ND: not determined, N Tot: total number of specimens collected, N Inv: number of sampled identified and processed for parasite identification, N Inf: number of specimens infected according to parasitological and molecular investigations, N Isol: number of isolates.

**Table 3:** Diagnostics of the 96 *Leishmania* isolates, using parasitological (Giemsa staining and in vitro culture) and molecular methods (PCR-RFLP and qPCR).

	Number of Samples		%		Sensitivity	
Test					%	IC
	Positive Case	Negative Case	Positive Case	Negative Case		
Smears	44	52	45.83	54.16	62	[50.7–73.3]
Culture	51	44	53.68	46.31	72.9	[62.4–83.3]
ITS1-PCR	54	40	57.44	42.55	78.3	[66.5–88.0]
RT-PCR	69	25	73.40	26.59	100	[94.8–100]
Total	71	25	73.95	26.04		

**Table 4:** Identification of sandfly species belonging to the *Phlebotomus* genus collected during the survey.

District	Station	*P. papatasi*	*P. perniciosus*	*P. alexandri*	*P. sergenti*	*P. chabaudi*	*P. longicuspis*	*P. perfiliewi*	*P. langeroni*	Total
M’Sila	Nouara	349	125	2	1	0	655	2	0	1134
	Boukhmissa	73	30	0	1	0	9	0	0	113
Chellal	Ouled Madhi	178	1	2	0	0	1	0	0	182
	Khettouti Sed El Djir	52	1	0	1	0	0	0	0	54
	Ait Ikhlef	60	5	0	0	2	3	0	0	70
Magra	Berhoum	23	6	0	0	0	1	0	0	30
	Ain El Khadra	6	0	0	0	0	0	0	0	6
	Dehahna	27	14	0	0	0	6	0	0	47
Hammam Dalaa	El Guetaf	28	3	2	0	0	12	1	0	46
Ouled Derradj	Maadid	28	199	3	36	0	29	0	0	295
Ain El Hadjel	Ain El Hadjel	2525	170	20	27	0	9	4	1	2756
Sidi Aissa	Sidi Aissa	39	4	0	0	0	0	0	0	43
Ain El Melh	Ain El Melh	13	0	0	0	0	0	0	0	13
Boussaâda	Boussaâda	3658	4	6	6	210	912	0	0	4796
	Total	7059	562	35	72	212	1637	7	1	9585

**Table 5:** Identification of sandfly species belonging to the *Sergentomyia genus* collected during the survey.

District	Station	*S. minuta*	*S. schwetzi*	*S. fallax*	*S. antennata*	*S. dreyfussi*	*S. lewisi*	*S. christophersi*	*S. clydei*	Total
M’Sila	Nouara	23	51	21	5	30	0	0	0	130
	Boukhmissa	5	1	0	53	0	0	0	1	60
Chellal	Ouled Madhi	40	67	355	263	1	0	0	0	726
	Khettouti Sed El Djir	0	0	0	28	0	0	0	0	28
	Ait Ikhlef	4	0	0	5	0	0	0	0	9
Magra	Berhoum	2	0	0	1	0	0	0	0	3
	Ain El Khadra	0	1	0	4	0	0	0	0	5
	Dehahna	1	0	0	12	0	0	0	0	13
Hammam Dalaa	El Guetaf	0	1	0	17	0	0	0	0	18
Ouled Derradj	Maadid	4	0	2	0	0	0	0	0	6
Ain El Hadjel	Ain El Hadjel	16	1	0	0	0	0	0	0	17
Sidi Aissa	Sidi Aissa	0	0	0	7	0	0	0	0	7
Ain El Melh	Ain El Melh	2	0	3	2	0	0	0	0	7
Boussaâda	Boussaâda	59	0	8	6	15	3	1	0	92
	Total	156	122	389	403	46	3	1	1	1121

**Table 6:** The infection rate of rodent samples as recorded by parasitological and molecular methods.

District	Family	Genus/Species	Year	N Inv	N Inf	Infestation Rate (%)	CI
Chellal, Sidi Aissa, other	*Gerbillidae*	*Psamomys obesus*	1996	-	-	-	
			2003	10	7	70	[38.01–91.74]
			2004	30	5	16.66	[6.37–33.15]
			2007	-	-	-	
			2010	144	43	29.86	[22.81–36.34]
			2018	12			
			Total	**186**	**55**	**29.57**	**[23.34–36.43]**
M’Sila, Sidi Aissa, Magra, Ain-El-Hadjel	*Gerbillidae*	*Meriones shawi*	1996	-	-		
			2000	-	-		
			2004	10	3	30	[8.26–61.99]
			2008	20	6	30	[13.16–52.28]
			2009	-	-		
			2012	6	1	16.66	[0.83–59.09]
			2018	1			
			Total	**37**	**10**	**27.02**	**[14.62–42.91]**

CI: Confidence Interval.

## Data Availability

Not applicable.
